# Supplementary material for: Establishment and validation of a prognostic nomogram for severe fever with thrombocytopenia syndrome: A retrospective observational study
Source: PLoS One. 2024 Oct 24;19(10):e0311924. doi: 10.1371/journal.pone.0311924 (PMC11500966; doi:10.1371/journal.pone.0311924)
Supplement: Attachment — Submitted filename: Response to Reviewers.docx [file pone.0311924.s002.docx]

Dear Dr. Wiyarta and Reviewers,

We would like to express our sincere appreciation for your thorough review and constructive comments on our manuscript titled “Establishment and validation of a prognostic nomogram for severe fever with thrombocytopenia syndrome: a retrospective observational study” We have carefully considered each point raised and have made the necessary revisions. Below, we provide detailed responses to each comment and outline the changes made in the manuscript.

**Response to Editor's Comments**

**Comment 1:** *Please ensure your manuscript complies with PLOS ONE's style requirements, including file naming conventions.*

**Response 1:** We have reviewed and updated our manuscript to align with the *PLOS ONE* template and style guidelines.

**Comment 2:** *Code and Data Availability Statement.*

**Response 2:** We have revised the data availability statement in our submission to ensure it meets *PLOS ONE*'s requirements.

**Comment 3:** *ORCID ID*

**Response 3:** We have added the ORCID ID for the corresponding author as requested.

**Response to Reviewer #1**

**Comment 1:** *Some parts of the manuscript exhibit a somewhat Chinese style of expression. It is recommended to polish the language throughout the manuscript to ensure correct grammar, clear expression, and compliance with academic writing standards.*

**Response 1:** We have polished the language throughout the manuscript to enhance clarity, correct grammar, and ensure it meets academic writing standards.

**Comment 2:** *In the results section of the abstract, in addition to the ROC value, other performance indicators (such as sensitivity, specificity, accuracy, etc.) can be mentioned to comprehensively demonstrate the superiority of the new nomogram in this study.*

**Response 2:** We have included additional performance indicators such as accuracy, sensitivity, specificity, precision, and F1 score in the results section of the abstract to provide a comprehensive demonstration of the model's performance.

**Comment 3:** *It is suggested to add a brief explanation of the nomogram prediction model in the results section, including how to use the model specifically, to help readers better understand the necessity of the study and verify its clinical practicality.*

**Response 3:** We have added a brief explanation of how to use the nomogram prediction model in the results section (line 200).

**Comment 4:** *Some details need modification, such as inconsistent capitalization of variables in Table 1.*

**Response 4:** The capitalization of variables in Table 1 has been standardized and corrected.

**Response to Reviewer #2**

**Comment 1:** *Hyperproduction of several cytokines such as interleukin-6 (IL-6), IL-8, IL-10, IL-1β, tumor necrosis factor (TNF-α), Interferon alpha (IFN-α), IFN-γ, and transforming growth factor‐β (TGF‐β) have been proved to correlate with fatal viral diseases such as SFTS patients. Could authors show cytokine data in this study?*

**Response 1:** In the introduction section, we discussed the association between cytokine levels and the severity of SFTS. However, cytokine data were not collected as part of this study. We acknowledge their potential importance in understanding disease mechanisms and prognosis. Future studies may explore these immunological factors to further refine predictive models and improve patient outcomes. Thank you for your interest in our research, and we look forward to addressing this aspect in future investigations.

**Comment 2:** *1) In line 38, SFTSV virus: Could the authors check this? SFTS virus or Severe Fever with Thrombocytopenia Syndrome Virus (SFTSV); 2) In line 43, the prevalence of SFTS is expected to rise with global warming: Could the authors put reference(s)?; 3) In line 49, APACHE II: Could the authors write the full name of APACHE II?; 4) In line 292, "the focus of studies about COVID-19 during the pandemic": Could authors also write the full name of COVID-19?*

**Response 2:**

1) In line 42, the term "SFTSV virus" has been corrected to "Severe fever with thrombocytopenia syndrome virus (SFTSV)".
2) A relevant reference has been added to support the statement in line 48 regarding the expected increase in SFTS prevalence due to global warming.
3) The full name of APACHE II, "Acute Physiology and Chronic Health Evaluation II," has been provided (line 57).
4) The full name of COVID-19, "Coronavirus Disease 2019," has been included (line 305).

We believe these revisions and responses adequately address the comments and suggestions provided. We appreciate your consideration and look forward to your feedback.

Sincerely,

Yishan Zheng
